# Assessment of clinical course among patients with major depressive disorder treated with sertraline: a retrospective observational real-world study

**DOI:** 10.1192/j.eurpsy.2025.470

**Published:** 2025-08-26

**Authors:** P. Purushottamahanti, C. U. Correll, K. Subramaniam, B. Bayan, N. Lipunova, E. O. Palmer, J. Yeow, J. Wong

**Affiliations:** 1 Global Medical Affairs, Viatris Inc., Bangalore, India; 2 Department of Psychiatry, Zucker Hillside Hospital, Northwell Health, Glen Oaks, NY; 3 Department of Psychiatry and Molecular Medicine, Zucker School of Medicine at Hofstra/Northwell, Hempstead, NY; 4 Center for Psychiatric Neuroscience, Feinstein Institute for Medical Research, Manhasset, NY, United States; 5 Department of Child and Adolescent Psychiatry, Charité Universitätsmedizin, Berlin, Germany; 6 Global Medical Affairs, Viatris Inc., Auckland, New Zealand; 7 GMARH, Viatris Inc., Bangalore, India; 8 Holmusk Technologies Inc., London, United Kingdom; 9 Holmusk Technologies Inc., Singapore, Singapore

## Abstract

**Introduction:**

Major depressive disorder (MDD) is a chronic condition with recurrence rates ranging between 50-90%. MDD is also one of the leading causes of functional impairment (Moriarty AS *et al*. *Br J Gen Pract.* 2020 Jan 30;70(691):54-55). Therefore, clinical practice guidelines (CPGs) recommend continuing antidepressant treatment of MDD (6-24 months) beyond achieving clinical remission to prevent relapse. Sertraline, a selective serotonin reuptake inhibitor, is recommend as a first line agent by most CPGs in the management of MDD (Lam RW *et al. Can J Psychiatry.* 2024 Sep;69(9):641-687, NICE guideline Jun 2022).

**Objectives:**

To evaluate the effectiveness of sertraline in the long-term management of MDD in a real-world clinical practice setting.

**Methods:**

A retrospective, observational study of real-world data assessed the clinical course in patients with MDD with or without comorbid anxiety disorders (N=713, female=526), who were prescribed sertraline. Data from >25 mental health centers in the USA from Holmusk’s NeuroBlu database were used to estimate the effectiveness of sertraline in patients with moderate to severe MDD (CGI-S ≥ 4, n=556, female=414). Changes in CGI-S from baseline to months 2, 3, 6, 9 and 12 following sertraline initiation were analyzed. One point or more reduction in CGI-S was regarded as improvement, one point or more CGI-S increase was by denoted as worsening. Stability was assumed where no CGI-S change was observed.

**Results:**

In comparison to baseline, patients showed a general trend for improvement, regardless of the available follow-up duration **(Fig 1)**. In total, 32.7% to 41.9% patients with moderate to severe MDD showed clinically meaningful improvement across any of the time points **(Fig 2)**.

**Image 1:**

**Figure:**
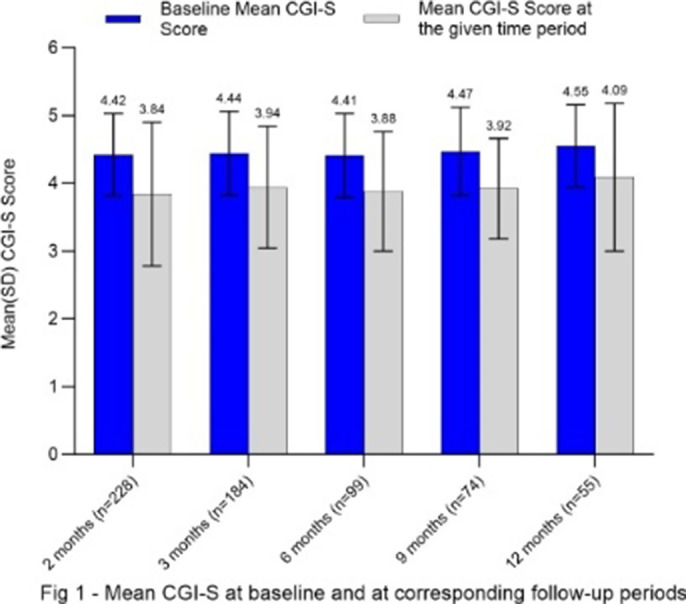

**Image 2:**

**Figure:**
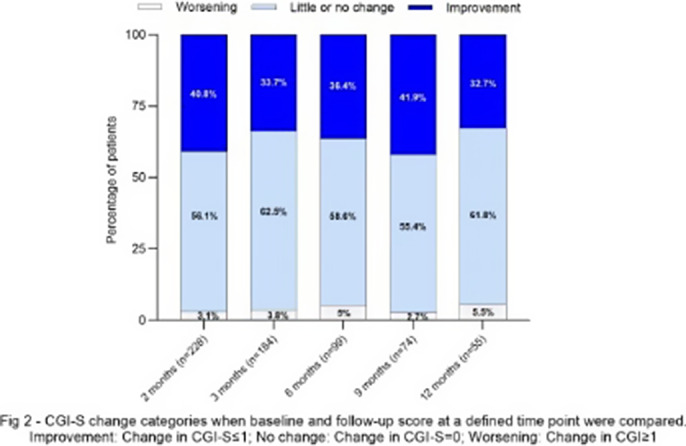

**Conclusions:**

The results from this retrospective analysis of health records suggest that sertraline is an effective treatment in the management of MDD in real-world clinical practice, even in the long-term.

**Limitations:**

First, this study only assessed patients who had CGI-S recorded at baseline and at least one additional recorded at any of the pre-defined follow-up points. As a result, patients who did not have a follow-up CGI-S value or had the value recorded outside of the pre-defined follow-up points were excluded from the study. The study also did not assess detailed MDD symptom ratings. Finally, information was lacking whether patients were treated previously in other clinical setting.

**Disclosure of Interest:**

P. Purushottamahanti Employee of: Viatris, C. Correll Shareolder of: Cardio Diagnostics, Kuleon Biosciences, LB Pharma, MedLink, Mindpax, Quantic, and Terran., Grant / Research support from: Bendheim Research Foundation, Boehringer-Ingelheim, German Ministry for Education and Research; German Research Foundation; Janssen, National Institute of Mental Health (NIMH), Patient Centered Outcomes Research Institute (PCORI), Takeda, Thrasher Foundation, Speakers bureau of: AbbVie, Acadia, Adock Ingram, Alkermes, Allergan, Angelini, Aristo, Biogen, Boehringer-Ingelheim, Bristol-Meyers Squibb, Cardio Diagnostics, Cerevel, CNX Therapeutics, Compass Pathways, Darnitsa, Delpor, Denovo, Gedeon Richter, Hikma, Holmusk, IntraCellular Therapies, Jamjoom Pharma, Janssen/J&J, Karuna, LB Pharma, Lundbeck, MedInCell, Merck, Mindpax, Mitsubishi Tanabe Pharma, Maplight, Mylan, Neumora Therapeutics, Neurocrine, Neurelis, Newron, Noven, Novo Nordisk, Otsuka, PPD Biotech, Recordati, Relmada, Reviva, Rovi, Sage, Saladax, Seqirus, SK Life Science, Sumitomo Pharma America, Sunovion, Sun Pharma, Supernus, Tabuk, Takeda, Teva, Tolmar, Vertex, Viatris and Xenon Pharmaceuticals., K. Subramaniam Employee of: Viatris, B. Bayan Employee of: Viatris, N. Lipunova Employee of: Holmusk Inc., E. Palmer Employee of: Holmusk Inc., J. Yeow Employee of: Holmusk Inc., J. Wong Employee of: Holmusk Inc.

